# Demographic, behavioral, and cardiovascular disease risk factors in the Saudi population: results from the Prospective Urban Rural Epidemiology study (PURE-Saudi)

**DOI:** 10.1186/s12889-020-09298-w

**Published:** 2020-08-08

**Authors:** Khalid F. Alhabib, Mohammed A. Batais, Turky H. Almigbal, Mostafa Q. Alshamiri, Hani Altaradi, Sumathy Rangarajan, Salim Yusuf

**Affiliations:** 1 Department of Cardiac Sciences, King Fahad Cardiac Center, College of Medicine, King Saud University, Riyadh, Saudi Arabia; 2 Department of Family and Community Medicine, College of Medicine, King Saud University, Riyadh, Saudi Arabia; 3 Population Health Research Institute, DBCVS Research Institute, McMaster University, Hamilton, Canada

**Keywords:** Demographics, Prevalence, Risk factors, Cardiovascular disease, Urban, Rural, Saudi Arabia

## Abstract

**Background:**

Cardiovascular disease (CVD) is the major cause of death in Saudi Arabia. We aimed to assess associated demographic, behavioral, and CVD risk factors as part of the Prospective Urban Rural Epidemiology (PURE) study.

**Methods:**

PURE is a global cohort study of adults ages 35–70 years in 20 countries. PURE-Saudi study participants were recruited from 19 urban and 6 rural communities randomly selected from the Central province (Riyadh and Alkharj) between February 2012 and January 2015. Data were stratified by age, sex, and urban vs rural and summarized as means and standard deviations for continuous variables and as numbers and percentages for categorical variables. Proportions and means were compared between men and women, among age groups, and between urban and rural areas, using Chi-square test and t-tests, respectively.

**Results:**

The PURE-Saudi study enrolled 2047 participants (mean age, 46.5 ± 9.12 years; 43.1% women; 24.5% rural). Overall, 69.4% had low physical activity, 49.6% obesity, 34.4% unhealthy diet, 32.1% dyslipidemia, 30.3% hypertension, and 25.1% diabetes. In addition, 12.2% were current smokers, 15.4% self-reported feeling sad, 16.9% had a history of periods of stress, 6.8% had permanent stress, 1% had a history of stroke, 0.6% had heart failure, and 2.5% had coronary heart disease (CHD). Compared to women, men were more likely to be current smokers and have diabetes and a history of CHD. Women were more likely to be obese, have central obesity, self-report sadness, experience stress, feel permanent stress, and have low education. Compared to participants in urban areas, those in rural areas had higher rates of diabetes, obesity, and hypertension, and lower rates of unhealthy diet, self-reported sadness, stress (several periods), and permanent stress. Compared to middle-aged and older individuals, younger participants more commonly reported an unhealthy diet, permanent stress, and feeling sad.

**Conclusion:**

These results of the PURE-Saudi study revealed a high prevalence of unhealthy lifestyle and CVD risk factors in the adult Saudi population, with higher rates in rural vs urban areas. National public awareness programs and multi-faceted healthcare policy changes are urgently needed to reduce the future burden of CVD risk and mortality.

## Background

Cardiovascular disease (CVD) is the leading cause of mortality worldwide, contributing to 31% of all deaths [1]. CVD is also becoming a major health concern in the Gulf Council Countries, including Saudi Arabia, where it is estimated that CVD accounts for more than 45% of all deaths [1, 2]. The most common CVD risk factors identified in the INTERHEART and INTERSTROKE studies were hypertension, diabetes, dyslipidemia, obesity, smoking, low physical activity, poor diet, and alcohol consumption [3, 4]. In the Gulf countries, lifestyle has changed dramatically because of rapid urbanization, with an increase in poor diet and the adoption of a sedentary lifestyle. Consequently, rates of CVD risk factors and chronic non-communicable diseases in the Gulf population are also high [2, 5–32].

The aim of the Prospective Urban Rural Epidemiology (PURE) cohort study is to collect data on social, environmental, and individual CVD risk factors and chronic diseases in high-, middle-, and low-income countries [33]. Saudi Arabia has been classified as a high-income country and joined the global PURE study in 2012. The current PURE Saudi report focuses mainly on assessing the demographics, unhealthy lifestyle, and prevalence of CVD risk factors, stratified by age, sex, and place of residence (urban vs rural). Given the relatively small sample size at this stage, we have reported only the absolute number of cases diagnosed with cancer, myocardial infarction, stroke, heart failure, and death during a median (interquartile range [IQR]) follow-up of 3.4 (3.2–6.1) years.

## Methods

### Study design and participants

The study design, methods (including sampling, information gathered, and follow-up strategy), and participant characteristics of the PURE study have been published previously [33–36]. Briefly, the study included adults ages 35 to 70 years from 367 urban and 302 rural communities in 20 countries. Households were eligible if one or more members was age 35 to 70 years and the household members intended to stay at that address for a further 4 years. Risk factors and medical history were documented, and a physical examination was performed on participants who provided written informed consent. Details of sampling, information gathered, and follow-up strategy have been previously reported [34, 35].

### Procedures

Data regarding demographic factors, socioeconomic status, medical history, health behaviors (e.g., smoking, physical activity, diet, alcohol intake), and household members living with participants were collected by using standardized questionnaires. Low education was defined as no education, primary education only, or unknown educational level. Diet quality was determined based on the Alternative Healthy Eating Index, with scores ranging from 6 to 70 and higher scores indicating more healthful eating [37, 38]. A score of less than 31 was taken as indicating an unhealthful diet. In addition, we collected information regarding psychosocial factors (feeling “blue” and general stress) and other CVD risk factors such as hypertension, diabetes, and obesity, as described in the INTERHEART study [3, 4, 39]. We defined stress as reporting stress over several periods and/or having permanent stress. Operationally, stress is defined as feeling irritable or anxious or having sleeping difficulties as a result of conditions at work or at home. We categorized stress as follows: 1 = none or little; 2 = moderate; and 3 = high–severe.

The questionnaire also asked about anthropometric measures, including weight, height, body mass index (BMI, kg/m^2^), waist circumference, and blood pressure. Physical activity was measured using the International Physical Activity Questionnaire and was categorized according to the metabolic equivalent of task (MET) per min per week as low activity at 600 or fewer MET min per week [40].

Participants were considered to have diabetes if they were previously diagnosed by a physician, had a fasting plasma glucose ≥126 mg/dl (7.0 mmol/l), or were being treated with glucose-lowering medication. Those with history of hypertension, current use of antihypertensive medication, and/or blood pressure ≥ 140 (systolic) or ≥ 90 (diastolic) mmHg were considered to have hypertension. All respondents were asked whether they had a medical diagnosis of hypertension (awareness) and whether they were receiving antihypertensive medication (treatment). Hypertension control was defined as the proportion of participants with an average systolic/diastolic blood pressure of < 140/90 mmHg. Following a standardized procedure provided for all sites, blood pressure was measured two times using an Omron digital blood pressure device (Omron HEM-757; Omron Healthcare, Kyoto, Japan) at the right arm, with the participant in a sitting position. A total cholesterol level > 5.2 mmol/l (201 mg/dl) was considered to be elevated. Major CVD (myocardial infarction or angina, stroke, or heart failure) was the main clinical outcome included in the analyses based on participant self-reported responses. We assessed CVD risk using the INTERHEART Risk score, which is a validated score for quantifying risk-factor burden without the use of laboratory testing. Scores range from 0 to 48, with higher scores indicating greater risk-factor burden.

### PURE Saudi

Recruitment of Saudi individuals for the PURE study was carried out between February 2012 and January 2015. Nineteen urban and six rural communities were enrolled from the city of Riyadh and Alkharj (Central province). Urban communities involved were defined according to the governmental geographic distribution of the districts, whereas rural communities were defined as those regions located at least 50 km from the center of Riyadh. For cultural acceptance, the study team met the participants in the primary healthcare centers (PHCCs) in each community. The database of each PHCC was screened, and eligible candidates were randomly invited to participate in the study through a call and/or short text message to their mobile phones. A target number of at least 50 participants was required to be enrolled through each PHCC. All participants were encouraged to invite their eligible family members living in the same household. All blood samples were shipped to the laboratory in the King Khalid University Hospital at King Saud University, Riyadh. Results of the blood tests were returned to the treating physicians at the PHCC for further assessment and management.

During the follow-up period, the study team placed a telephone call at 18 months after the baseline recruitment to remind participants about their upcoming follow-up at 3, 6, and 9 years. During a median (IQR) follow-up of 3.4 (3.2–6.1) years, the study team recorded death, cancer, myocardial infarction, stroke, and heart failure based on self-reports from the participant or a family member. Incidence rates were then calculated, taking into account the duration of follow-up and time to the corresponding events.

### Statistical analysis

The total number of participants recruited for this study was determined using convenience sampling based on real-life acceptance rates from the community, with the aim also of having a reasonable representation of participants from across communities in the Riyadh region. We summarized categorical variables using frequencies and percentages, and for continuous variables, we used means and standard deviations (SDs) or medians and IQRs. To compare proportions of participants in different groups, we used the Chi-square or Fisher’s exact test for categorical variables, the student’s t-test or Mann–Whitney U test for continuous variables between two groups, and analysis of variance or the Kruskal–Wallis test for continuous variables among more than two groups. Age was categorized into three groups: 35–49 years, 50–59 years, and 60–70 years. Education was categorized as high (i.e., trade school, college, or university), medium (i.e., secondary school or high school), low (i.e., primary education or no education), or unknown. We also calculated the incidence rate of events taking into account follow-up duration and time to the corresponding events in 100-person years*.* All statistical analyses were performed using SAS version 9.2 (SAS Institute, Inc., Cary, NC, USA). A *P* value of less than 0.05 was considered statistically significant.

### Human participant protection

The King Saud University Ethics Committee granted ethics approval for the study. Participation in the study was voluntary, and all eligible participants who provided written informed consent were enrolled.

## Results

### Overall cohort

The PURE-Saudi study enrolled 2047 participants, with a mean age of 46.5 ± 9.12 years (Table 1). There were 1165 men (56.9%) and 882 women (43.1%). Around one third of the total cohort had a low educational level.

**Table 1:** Characteristics and the prevalence of cardiovascular disease risk factors in the PURE-Saudi study

Characteristics	Overall	Men	Women	*P*^1^
N (%)	2047	1165 (56.9)	882 (43.1)	
Demographics				
Age (y), mean ± SD	46.5 ± 9.1	47.5 ± 9.4	45.1 ± 8.5	< 0.001
Low educational level, n (%)	646 (31.6)	235 (20.2)	411 (46.6)	< 0.001
Behavioral risk factors				
Smoking status, n (%)				
Current smoker	249 (12.2)	245 (21)	4 (0.4)	< 0.001
Former smoker	217 (10.6)	209 (17.9)	8 (0.9)	< 0.001
Unhealthful diet, n (%)	702 (34.4)	397 (56.5)	305 (43.4)	0.827
Low physical activity, n (%)	1415 (69.4)	805 (69.3)	610 (69.5)	0.946
Current alcohol use, n (%)	24 (1.2)	24 (2.1)	0 (0.0)	< 0.001
Hypertension				
Hypertension, n (%)	620 (30.3)	382 (32.8)	238 (27)	0.005
Awareness among patients with known hypertension, n (%)	379 (61.1)	233 (61)	146 (61.3)	0.931
Treated hypertension among patients with known hypertension, n (%)	365 (58.9)	220 (57.6)	145 (60.9)	0.412
Controlled hypertension among those with known hypertension, n (%)	190 (30.6)	108 (28.3)	82 (34.4)	0.104
Treated hypertension and SBP ≥140 mmHg and/or DBP ≥90 mmHg, n (%)	175 (47.9)	112 (50.9)	63 (43.4)	0.163
Treated hypertension and SBP > 120 mmHg and/or DBP > 80 mmHg, n (%)	318 (87.1)	198 (90)	120 (82.8)	0.043
Treated hypertension and SBP > 130 mmHg and/or DBP > 80 mmHg, n (%)	272 (76.2)	173 (79.4)	99 (71.2)	0.078
Diabetes				
Diabetes, n (%)	516 (25.2)	328 (28.1)	188 (21.3)	< 0.001
Among patients with diabetes				0.093
On insulin alone	14 (2.7)	5 (1.5)	9 (4.8)	
On OHA alone	313 (60.7)	200 (61)	113 (60.1)	
On both	34 (6.6)	19 (5.8)	15 (8)	
Not on prescription drug	155 (30)	104 (31.7)	51 (27.1)	
Dyslipidemia				
Total cholesterol > 5.2 mmol/l and low-density lipoprotein cholesterol > 3.5 mmol/l, n (%)	569 (32.1)	234 (31.2)	335 (32.7)	0.508
Obesity				
BMI, mean	30.6 ± 5.9	29.7 ± 5.4	31.9 4 ± 6.3	< 0.001
BMI, n (%)				< 0.001
< 25	310 (15.1)	205 (17.6)	105 (11.9)	
25–30	722 (35.3)	462 (39.7)	260 (29.5)	
31–35	613 (30)	463 (39.8)	286 (32.4)	
> 35	399 (19.5)	169 (14.5)	230 (26.1)	
Abdominal obesity, n (%)				
Waist circumference > 102 cm (men) or > 88 cm (women)	1005 (49)	381 (32.7)	624 (70.7)	< 0.001
Waist circumference > 90 cm (men) or > 85 cm (women)	1521 (74.3)	835 (71.7)	686 (77.8)	0.002
Psychosocial				
Self-report of feeling sad or “blue,” n (%)	315 (15.4)	115 (9.9)	200 (22.7)	< 0.001
General feeling of stress, n (%)				
Several periods of stress	339 (16.9)	143 (12.4)	196 (23.1)	< 0.001
Permanent stress	136 (6.8)	52 (4.5)	84 (9.9)	< 0.001
Medical history				
History of ischemic heart disease (angina, myocardial infarction, or any coronary revascularization), n (%)	51 (2.5)	37 (3.2)	14 (1.6)	0.022
History of stroke, n (%)	20 (1)	12 (1)	8 (0.9)	0.779
History of heart failure, n (%)	13 (0.6)	5 (0.4)	8 (0.9)	0.178
INTERHEART Risk Score, median (25th–75th, IQR)	11 (8,16)	13 (9,18)	10 (6,14)	< 0.001

*BMI* body mass index, *DBP* diastolic blood pressure, *IQR* interquartile range, *OHA* oral hypoglycemic agent, *SBP* systolic blood pressure

^1^*P* values refer to the results of either Chi-square tests (for categorical variables) or t-tests (for continuous variables comparing the mean between categories

### CVD risk factors

The mean BMI of the participants was 30.6, with the majority either overweight (35.3%) or obese (49.6%). Among patients with obesity, 30% had a BMI of 30–35, and 19.5% had a BMI > 35. The prevalence of abdominal obesity, defined as a waist circumference > 102 cm (men) or > 88 cm (women), was 49%. This prevalence increased to 74.3% when the waist circumference cutoff was > 90 cm (men) or > 85 cm (women).

The prevalence of hypertension was 30.3%, of whom only 61.1% were aware of it, 58.9% were treated, and 30.7% had achieved blood pressure control. The prevalence of diabetes was 25.1%, and of these participants, 2.7% were on insulin therapy, 60.7% received oral hypoglycemic agents (OHAs), 6.6% received both (insulin and OHAs), and 30% received no treatment. About one third (32.1%) had a high total cholesterol level, and 1% had a history of stroke, 0.6% a history of heart failure, and 2.5% a history of coronary heart disease. The median INTERHEART risk score for the total cohort was 11.

### Health behaviors and psychosocial factors

Approximately 34.4% of the total cohort reported eating an unhealthy diet, 69.4% reported low physical activity, 12.2% were current smokers, and 10.6% were former smokers. Moreover, the prevalence of self-reported sadness or feeling blue was 15.4%, whereas 16.9% reported a history of feeling stress during several periods, and 6.8% had a permanent feeling of stress.

### Men compared with women

**Fig. 1:**
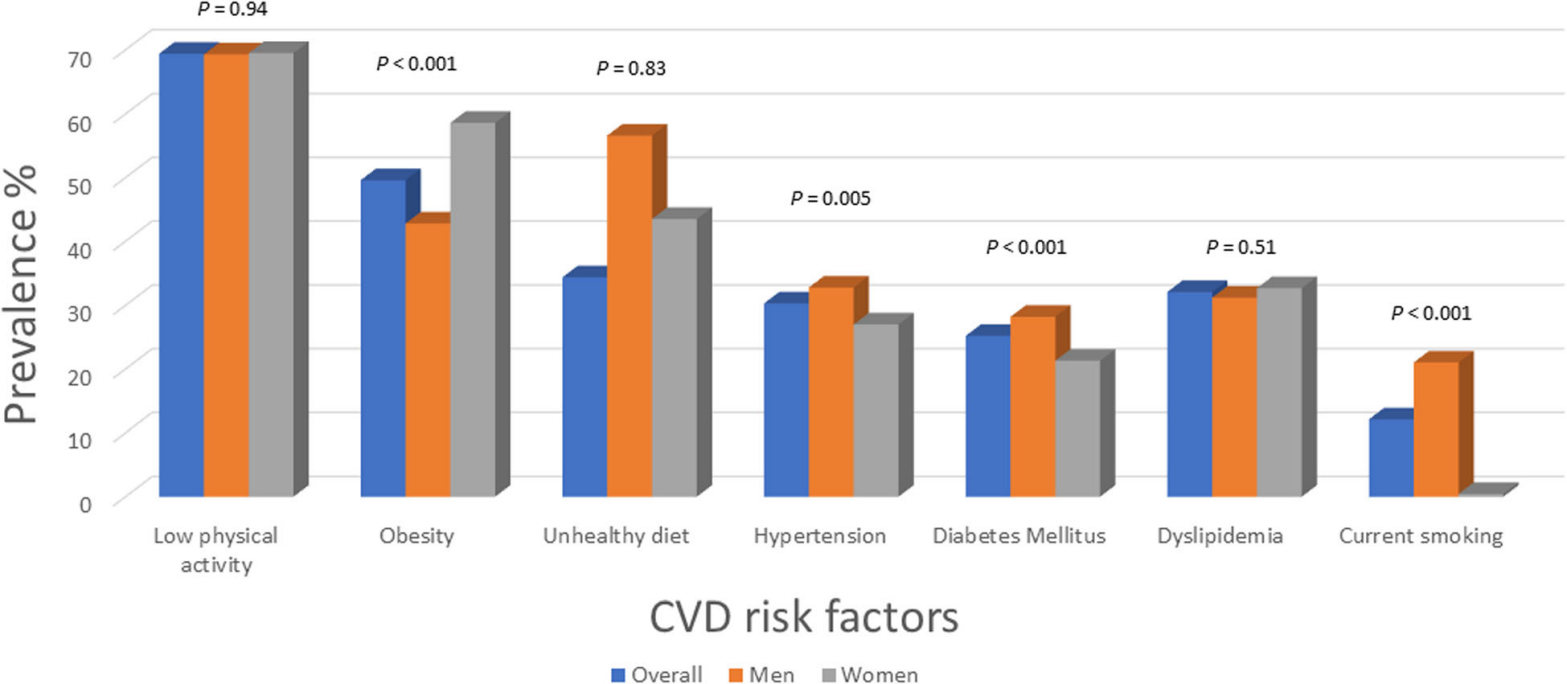
Prevalence of cardiovascular risk factors in the overall cohort and stratified by sex

Compared with women, men had a significantly higher proportion of current smoking (21% vs 0.45%, *P* < 0.001) or former smoking (17.9% vs 0.91%, *P* < 0.001), diabetes (28.2% vs 21.3%, *P* < 0.001), obesity with BMI 30–35 (39.9% vs 32.4%, *P* < 0.001), and history of ischemic heart disease (3.2% vs 1.6%, *P* = 0.02), and a higher median INTERHEART risk score (13% vs 10%, *P* < 0.001). Women had higher prevalence of obesity with BMI > 35 (26.1% vs 14.5%, *P* < 0.001), central obesity (70.7% vs 32.7%, *P* < 0.001), self-reported sadness (22.7%, vs 9.9%, *P* < 0.001), several periods of stress (23.1% vs 12.4%, *P* < 0.001), a permanent feeling of stress (9.9% vs 4.5%, *P* < 0.001), and low level of education (46.6% vs 20.2%, *P* < 0.001) (Table 1 and Fig. 1).

Regarding blood pressure, for women compared with men, awareness (61.3% vs 60.9%, *P* = 0.93), treatment (60.9% vs 57.6%, *P* = 0. 41), and control (34.5% vs 28.3%, *P* = 0.10) were similar. Men were more likely, however, to have lower high-density lipoprotein cholesterol levels compared with women (Additional Table 1).

### Young vs middle vs old age

**Fig. 2:**
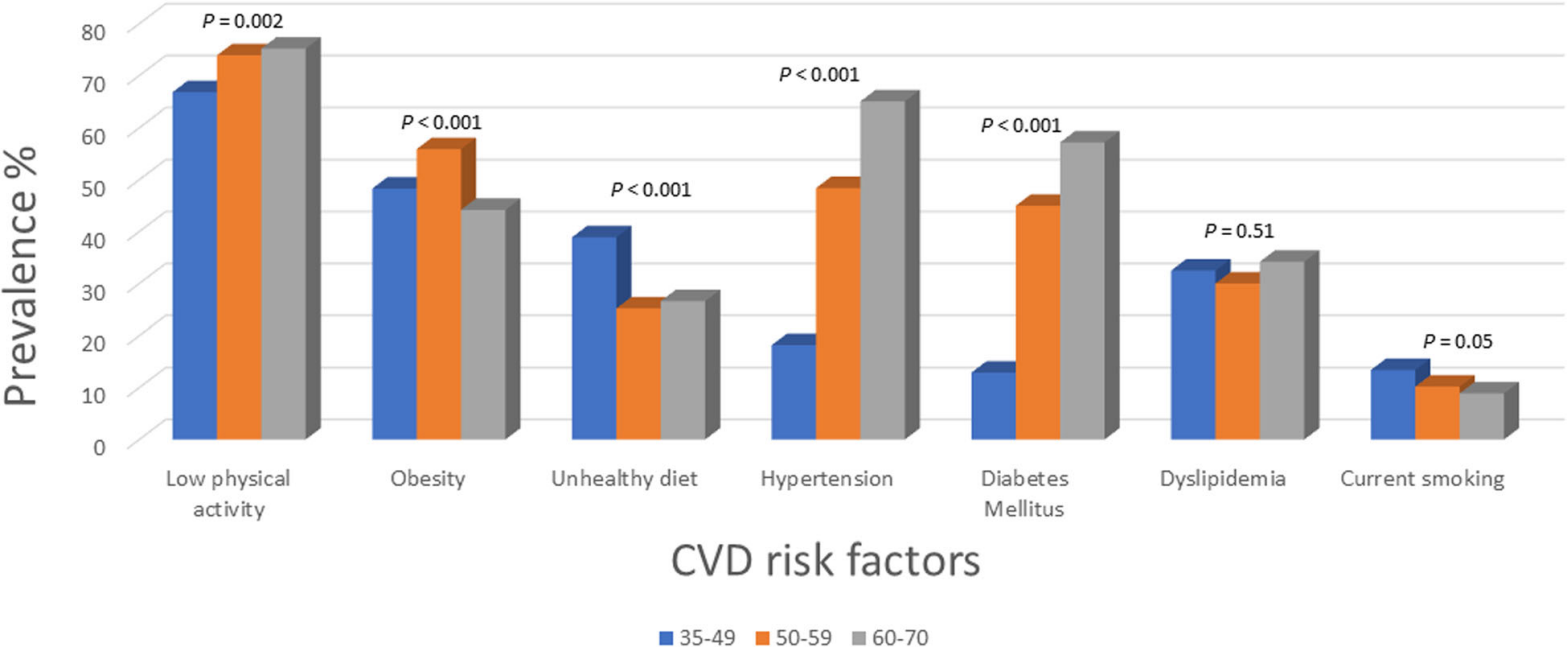
Cardiovascular risk factors stratified by age groups

Compared to the younger and middle age groups (35–49 years and 50–59 years), older participants (60–70 years) had a higher prevalence of low physical activity (66.8% younger vs 73.9% middle vs 75.2% older, *P* = 0.002), hypertension (18.2% vs 48.4% vs 65%, *P* < 0.001), diabetes (12.9% vs 45% vs 57.5%, *P* < 0.001), low education level (21.7% vs 45% vs 62.8%, *P* < 0.001), stroke (0.4% vs 1.1% vs 4%, *P* < 0.001), history of heart failure (0.1% vs 1.5% vs 1.8%, *P* < 0.001), and history of ischemic heart disease (1.1% vs 4.1% vs 7.5%, *P* < 0.001) (Table 2 and Fig. 2). Awareness and treatment of blood pressure were higher among older participants compared with middle-aged and younger individuals (awareness: 45.5% for younger vs 70% middle-aged vs 73.5% older; 40.7% vs 69.2% vs 73.5% for treatment; *P* < 0.0001 for all).

**Table 2:** Prevalence of cardiovascular disease risk factors stratified by age groups in the PURE-Saudi study

Characteristics	35–49 y	50–59 y	60–70 y	*P*^1^
N (%)	1352 (66)	469 (22.9)	226 (11)	
Demographics				
Low education level, n (%)	293 (21.7)	211 (45)	142 (62.8)	< 0.001
Behavioral risk factors				
Smoking status, n (%)				
Current smoker	181 (13.4)	48 (10.2)	20 (8.8)	0.054
Former smoker	124 (9.2)	60 (12.8)	33 (14.6)	0.011
Unhealthful diet, n (%)	524 (38.9)	118 (25.2)	60 (26.5)	< 0.001
Low physical activity, n (%)	899 (66.8)	346 (73.9)	170 (75.2)	0.002
Current alcohol use, n (%)	14 (1)	7 (1.5)	3 (1.3)	0.632
Hypertension				
Hypertension, n (%)	246 (18.2)	227 (48.4)	147 (65)	< 0.001
Awareness among patients with known hypertension, n (%)	112 (45.5)	159 (70)	108 (73.5)	< 0.001
Treated hypertension among patients with known hypertension, n (%)	100 (40.6)	157 (69.2)	108 (73.5)	< 0.001
Controlled hypertension among those with known hypertension, n (%)	49 (19.9)	89 (39.2)	52 (35.4)	< 0.001
Treated hypertension and SBP ≥140 mmHg and/or DBP ≥90 mmHg, n (%)	51 (51)	68 (43.3)	56 (51.8)	0.304
Treated hypertension and SBP > 120 mmHg and/or DBP > 80 mmHg, n (%)	86 (86)	135 (86)	97 (89.8)	0.609
Treated hypertension and SBP > 130 mmHg and/or DBP > 80 mmHg, n (%)	80 (80.8)	115 (73.7)	77 (75.5)	0.423
Diabetes				
Diabetes, n (%)	175 (12.9)	211 (45)	130 (57.5)	< 0.001
Among patients with diabetes				0.831
On insulin alone	5 (2.9)	6 (2.8)	3 (2.3)	
On OHA alone	105 (60)	130 (61.6)	78 (60)	
On both	12 (6.9)	10 (4.7)	12 (9.2)	
Not on prescription drug	53 (30.3)	65 (30.8)	37 (28.5)	
Dyslipidemia				
Total cholesterol > 5.2 mmol/l and low-density lipoprotein cholesterol > 3.5 mmol/l, n (%)	380 (32.4)	124 (29.9)	65 (34.2)	0.514
Obesity				
BMI, mean	30.6 ± 6	31.4 ± 5.5	29.5 ± 5.7	0.003
BMI, n (%)				< 0.001
< 25.0	211 (15.6)	46 (9.8)	53 (23.4)	
25.0–30.0	488 (36.1)	161 (34.3)	73 (32.3)	
30.1–35.0	388 (28.7)	160 (34.1)	65 (28.8)	
> 35.0	262 (19.4)	102 (21.7)	35 (15.5)	
Abdominal obesity, n (%)				
Waist circumference > 102 cm (men) or > 88 cm (women)	628 (46.4)	262 (55.9)	115 (50.9)	0.002
Waist circumference > 90 cm (men) or > 85 cm (women)	960 (71)	389 (82.9)	172 (76.1)	< 0.001
Psychosocial				
Self-report of being sad or “blue,” n (%)	252 (18.6)	47 (10)	16 (7.1)	< 0.001
General feeling of stress, n (%)				
Several periods of stress	249 (18.6)	74 (16.3)	16 (7.5)	< 0.001
Permanent stress	121 (9.1)	11 (2.4)	4 (1.9)	< 0.001
Medical history				
History of ischemic heart disease (angina or myocardial infarction), n (%)	15 (1.1)	19 (4.0)	17 (7.5)	< 0.001
History of stroke, n (%)	6 (0.4)	5 (1.1)	9 (4)	< 0.001
History of heart failure, n (%)	2 (0.1)	7 (1.5)	4 (1.8)	< 0.001
INTERHEART Risk Score, median (25th–75th, IQR)	10 (7,14)	14 (10,19)	16 (12,21)	< 0.001

*BMI* body mass index, *DBP* diastolic blood pressure, *IQR* interquartile range, *OHA* oral hypoglycemic agent, *SBP* systolic blood pressure

^1^*P* values refer to the results of either chi-square tests (for categorical variables) or analysis of variance (for continuous variables comparing the mean across categories)

A greater proportion of middle-aged participants (39.2%) had more controlled blood pressure compared to younger (19.9%) and older (35.4%) individuals (*P* < 0.0001). However, the younger age group more often reported consumption of an unhealthy diet (38.9% vs 25.2% middle-aged vs 26.5% older respondents, *P* < 0.001), general feeling of permanent stress (9.1% vs 2.4% vs 1.9%, *P* < 0.001), and being sad (18.6% vs 10% vs 7.1%, *P* < 0.001). The prevalence of obesity with BMI 30–35 and BMI > 35 was 34.1 and 21.8% in the middle-aged group compared to 28.7 and 19.4% in the younger group and 28.8 and 15.5% in older age groups (*P* < 0.001) respectively. Moreover, the median level of glucose and triglycerides was significantly increased in the older age group (Additional Table 2).

### Urban vs rural

**Fig. 3:**
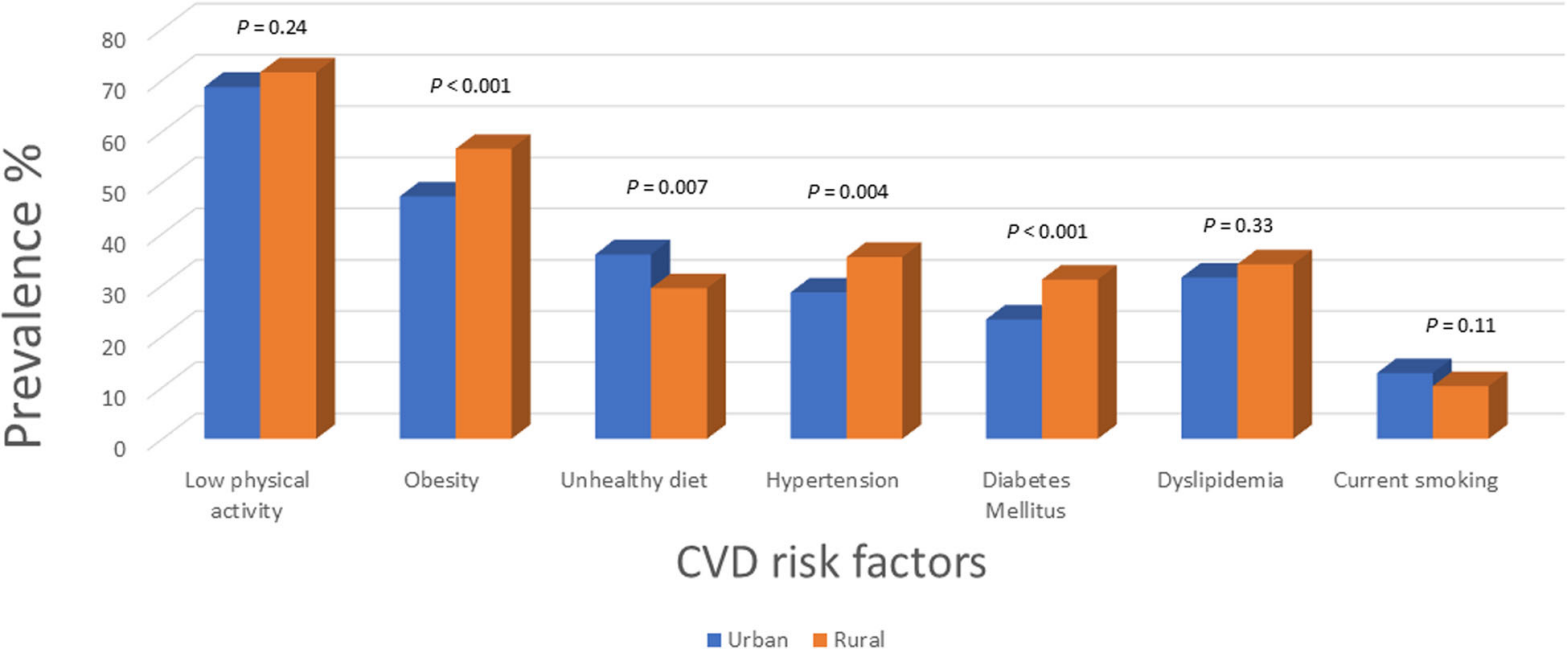
Cardiovascular risk factors stratified by rural vs urban residence

Of the cohort, 75.48% lived in urban areas. Compared to those in urban areas, a greater proportion of those living in rural areas reported diabetes (31.1% rural vs 23.3% urban, *P* < 0.001), obesity (56.6% vs 47.3%, *P* < 0.001), hypertension (35.5% vs 28.6%, *P* = 0.004), and low education level (46.4% vs 26.7%, *P* < 0.001). In addition, the median BMI was higher among rural compared to urban participants (30.8% vs 29.7%, *P* < 0.001). On the other hand, urban participants were more likely to consume unhealthy diet (36% vs 29.4%, *P* = 0.007), self-report being sad (17.6% vs 8.6%, *P* < 0.001), have several periods of stress (19.7% vs 8.7%, *P* < 0.001), and have a permanent feeling of stress (8.03% vs 3.02%, *P* < 0.001) (Table 3 and Fig. 3).

**Table 3:** Prevalence of cardiovascular disease risk factors stratified by place of residence in the PURE-Saudi study

Characteristics	Urban	Rural	*P*^1^
N (%)	1545 (75.5)	502 (24.5)	
Demographics			
Age (y), mean ± SD	46.3 ± 8.9	47.1 ± 9.8	0.124
Low education level, n (%)	413 (26.7)	233 (46.4)	< 0.001
Behavioral risk factors			
Smoking status, n (%)			
Current smoker	198 (12.8)	51 (10.2)	0.114
Former smoker	175 (11.3)	42 (8.4)	0.061
Unhealthful diet, n (%)	556 (36)	146 (29.4)	0.007
Low physical activity, n (%)	1061 (68.7)	354 (71.5)	0.240
Current alcohol use, n (%)	18 (1.2)	6 (1.2)	0.956
Hypertension			
Hypertension, n (%)	442 (28.6)	178 (35.5)	0.004
Awareness among patients with known hypertension, n (%)	275 (62.2)	104 (58.4)	0.381
Treated hypertension among patients with known hypertension, n (%)	266 (60.2)	99 (55.6)	0.296
Controlled hypertension among those with known hypertension, n (%)	144 (32.6)	46 (25.8)	0.100
Treated hypertension and SBP ≥140 mmHg and/or DBP ≥90 mmHg, n (%)	122 (45.9)	53 (53.5)	0.192
Treated hypertension and SBP > 120 mmHg and/or DBP > 80 mmHg, n (%)	227 (85.3)	91 (91.9)	0.095
Treated hypertension and SBP > 130 mmHg and/or DBP > 80 mmHg, n (%)	191 (73.5)	81 (83.5)	0.047
Diabetes			
Diabetes, n (%)	360 (23.3)	156 (31.1)	< 0.001
Among patients with diabetes			< 0.001
On insulin alone	11 (3.1)	3 (1.9)	
On OHA alone	249 (69.2)	64 (41.0)	
On both	31 (8.6)	3 (1.9)	
Not on prescription drug	69 (19.2)	86 (55.1)	
Dyslipidemia			
Total cholesterol > 5.2 mmol/l and low-density lipoprotein cholesterol > 3.5 mmol/l, n (%)	439 (31.5)	130 (34.1)	0.330
Obesity			
BMI, mean	30.4 ± 5.8	31.4 ± 6.2	0.037
BMI, n (%)			< 0.001
< 25.0	245 (15.9)	65 (12.9)	
25.0–30.0	569 (36.8)	153 (30.5)	
30.1–35.0	454 (29.4)	159 (31.7)	
> 35.0	275 (17.8)	124 (24.7)	
Abdominal obesity, n (%)			
Waist circumference > 102 cm (men) or > 88 cm (women)	754 (48.8)	251 (50)	0.641
Waist circumference > 90 cm (men) or > 85 cm (women)	1156 (74.8)	365 (72.7)	0.347
Psychosocial			
Self-report of being sad or “blue,” n (%)	272 (17.6)	43 (8.6)	< 0.001
General feeling of stress, n (%)			
Several periods of stress	296 (19.6)	43 (8.7)	< 0.001
Permanent stress	121 (8)	15 (3)	< 0.001
Medical history			
History of ischemic heart disease (angina or myocardial infarction), n (%)	36 (2.3)	15 (3)	0.411
History of stroke, n (%)	12 (0.8)	8 (1.6)	0.118
History of heart failure, n (%)	8 (0.5)	5 (1)	0.327
INTERHEART Risk Score, median (25th–75th, IQR)	11 (8,16)	12 (7,17)	0.833

*BMI* body mass index, *DBP* diastolic blood pressure, *IQR* interquartile range, *OHA* oral hypoglycemic agent, *SBP* systolic blood pressure

^1^*P* values refer to the results of either Chi-square tests (for categorical variables) or t-tests (for continuous variables comparing the mean between categories

The awareness, treatment, and control of blood pressure were similar between the urban and rural communities (awareness: 62.2% urban vs 58.4% rural, *P* = 0.38; treatment: 60.1% vs 55.6%, *P* = 0.29; control: 32.6% vs 25.8%, *P* = 0.10). Furthermore, urban participants had a higher median glucose level and higher proportion with a glucose level of 6–7 mmol/l in participants without diabetes (Additional Table 3).

### Follow-up

Of 2047 participants, 1996 responded to follow-up after baseline, for a response rate of 97.5%. Current mean (SD) and median (IQR) duration of follow-up is 4.3 (1.4) and 3.4 (3.2–6.1) years, respectively. We have attempted to contact participants at least three times. During the current follow-up, 6 patients had been diagnosed with cancer (0.07 per 100 person years of follow-up), and there were 22 deaths (0.26 per 100 person years of follow-up). The overall rates of major cardiovascular events showed a pattern similar to that for mortality: 24 had a myocardial infarction (0.29 per 100 person years of follow-up), 6 had a stroke (0.07 per 100 person years of follow-up), 4 had heart failure (0.05 per 100 person years of follow-up), and 34 had at least one major cardiovascular event (0.41 per 100 person years of follow-up).

## Discussion

### Main findings

The PURE-Saudi study allows for assessment of the prevalence of unhealthy lifestyle behaviors and CVD risk factors, stratified by age, sex, and place of residence. Because the work relies on internationally standardized and validated surveys of the global PURE study, valid interpretation and direct comparison of the results are possible in the context of other enrolled countries with variable economic scales and healthcare systems. Our study yielded two major findings. First, CVD risk factor prevalence is high in the Saudi population, including two thirds with low physical activity, half with obesity, one third who consume an unhealthy diet, one third with dyslipidemia, one third with hypertension, and one quarter with diabetes. Second, the relative proportion of the individual CVD risk factors varies with age, sex, and urban vs rural residence.

### CVD risk factor prevalence in comparison with previous findings

The PURE-Saudi study confirms that the prevalence of unhealthy life styles and coronary artery disease risk factors remains high in the Saudi population. These findings are in keeping with a decade of several previous population cross-sectional surveys (Additional Table 4) showing a high prevalence of hypercholesterolemia, obesity, hypertension, diabetes, smoking, physical inactivity, and diabetes in Saudi Arabia. The Coronary Artery Disease in the Saudis Study was a national epidemiological health survey conducted between 1995 to 2000 that included 17,395 Saudis ages 30–70 years through a multistage stratified cluster sampling technique. The overall prevalences were 54% for hypercholesterolemia, 35.6% for obesity, 26.1% for hypertension, 23.7% for diabetes, and 12.8% for smoking [11–13, 23–25]. Another national cross-sectional survey by the Saudi Ministry of Health involved 4758 participants ages 15 to 64 years and aimed to estimate the prevalence of some risk factors for non-communicable diseases by using the World Health Organization’s STEPwise approach for non-communicable disease surveillance. These results indicated prevalences of 67.6% for physical inactivity, 36.2% for obesity, 19.1% for hypercholesterolemia, 11.6% for hypertension, 15.3% for diabetes, and 10.9% for current daily smoking [14]. The Saudi Health Information Survey, also conducted by the Ministry of Health, enrolled 10,735 Saudis ages 15 years or older in 2013. The prevalences were 39.8% for low physical activity, 28.7% for obesity, 61.6% for daily consumption of < 2 servings of fruits and vegetables, 15.2% for hypertension, 13.4% for diabetes, 8.5% for hypercholesterolemia, and 12.1% for smoking [9, 10, 28, 29, 41]. As a result of such a high prevalence of CVD risk factors, patients in Saudi Arabia present almost a decade younger than average for developed countries with acute coronary syndromes and acute heart failure and thus are at high risk for cardiovascular complications and mortality [42–44].

### Diabetes and hypertension

Compared with the general population, patients with diabetes are 2 to 4 times more likely to develop CVD [45]. According to the International Diabetes Federation Diabetes Atlas (8th edition), Saudi Arabia is among the top 10 countries in diabetes prevalence [46], which is estimated to increase by 110% in the Middle East and North Africa by 2045. The prevalence in PURE Saudi was among the highest levels reported in the global PURE data. Overall, diabetes prevalence was 11%, varying among country income groups, with the lowest value (6.6%) in high-income countries and the highest (12.3%) in low-income countries [47]. Moreover, analysis of the global PURE data showed that hypertension prevalence was 40.8% and that only a third of patients had reached their blood pressure targets [48]. A report of prevalence, awareness, treatment, and control of hypertension from baseline PURE data from four Middle East countries [Iran, Occupied Palestinian Territory (OPT), Saudi Arabia, and the United Arab Emirates (UAE)] showed that one third had hypertension, about half of whom were aware and treated, and only one fifth were controlled [49]. The prevalence of hypertension was highest in the UAE (52%) and lowest in Iran (28%), whereas awareness, treatment, and control of hypertension were higher in the OPT and Saudi Arabia compared with the UAE and Iran [49].

Other national studies have found suboptimal hypertension control in the Saudi population [7, 24]. Affordability of medications is one of the main reasons for the low rate of hypertension control globally [50], but in Saudi Arabia, healthcare and medications are free, making them largely accessible to the population. Patients, physicians, and healthcare systems face several barriers in pursuing control of hypertension, indicating the need for multifaceted interventions [51, 52]. Forgetting to take medical therapies and medication side effects are important barriers to adherence [52]. Analysis of data from a large household survey of 10,735 participants to identify barriers to healthcare in Saudi Arabia found that neither distance to nor type of healthcare clinic were barriers to management of chronic diseases and highlighted the importance of an individual’s healthcare-seeking practices rather than system-related factors as potential barriers. It is possible that some in the Saudi population have specific healthcare-seeking practices that involve their seeking healthcare primarily when they feel unwell, contradicting the concept of an old Arab proverb, “Prevention is better than treatment” [53]. A lack knowledge and awareness of hypertension guidelines among primary care physicians also has been reported, with one survey finding that one fourth of 322 primary care physicians had deficient knowledge about the correct definition of hypertension [54]. Regarding the healthcare system barriers, inappropriate coordination among medical sectors has been reported, with about one third of Saudi patients with hypertension not having a record of the diagnosis on file at the primary healthcare centers and receiving medical care in different healthcare sectors that led to missing their regular appointments [51].

### Obesity

The overall prevalence of obesity in this study was approximately 49.6%, which was higher than the most recent national surveys conducted in Saudi Arabia, [9, 55] indicating the lack of effect of obesity prevention programs in the kingdom. In addition, a number of risk factors have been correlated with the increased prevalence of obesity in the country, including sedentary lifestyle and increasing trend in energy intake [56].

Our findings support those of a secondary analysis of published data that estimated trends and projections in the age- and sex-specific prevalence of adult obesity in Saudi Arabia over the 30-year period of 1992–2022 [55]. The prevalence of obesity was projected to increase markedly (by more than 200%) among men and women aged 25–64 years, with women having much higher projected prevalences than men [55]. One of the main findings of PURE Saudi is that women are more obese compared to men. A possible explanation of this higher obesity prevalence among women in this cohort may be sociocultural factors and governmental bylaws. These factors include the requirement until recently that women have a driver for transportation purposes, along with barriers to engaging in physical activities in public places. Increasing women’s access to exercise facilities and providing safe walking areas are likely to help to reduce the obesity prevalence. Recently, gymnasiums for women in Saudi Arabia have become more accessible, and women are now allowed to drive by themselves, which could potentially improve access to a healthier lifestyle. On the other hand, the lower rate of diabetes in women compared with men in our study may be attributed to the well-recognized greater willingness of women than men to seek medical advice [57]. In addition, women are also more willing than men to adhere to daily management of diabetes, such as restricted diet, blood glucose monitoring, and prescribed medication [58].

### Urban vs rural

Another important issue to highlight is the common belief that the risk of developing CVD is higher in individuals living in urban vs rural areas [59]. Findings from the global PURE cohort from high-income countries reported a similar INTERHEART risk score between populations in rural and urban areas [35]. However, the PURE Saudi study showed that the rural population had higher prevalence of CVD risk factors, particularly diabetes, hypertension, and obesity, compared to the urban population. Possible reasons might be related to what some researchers have called the “urbanization of rural life” [60], in which agriculture has become mechanized and cars are used for transport, road infrastructure has improved, and consumption of processed carbohydrates and commercially prepared and processed food has increased through the efforts of national and transnational companies, all of which would contribute to increased obesity [61–63]. In addition, the limited time and space for cooking healthy meals and possibly perceptions of greater weight as a sign of affluence could exacerbate these effects [63, 64]. Furthermore, our findings might reflect less access to and/or low availability of a healthcare prevention and management facilities in rural areas. The reasons for these disparities may include inconsistent insurance policies, poor healthcare infrastructure and privatization, and accessibility to healthcare facilities that largely focus on the urban population, leaving the rural population disadvantaged [65]. The higher prevalence of diabetes in rural rather than urban areas provides support for the association between diabetes and lifestyle risk factors because lifestyle changes are less prominent in rural areas. In addition, the lower educational level among the rural population identified in this study could in part explain the differences in risk factor levels, similar to findings of the Vasterbotten Intervention Program study in Sweden [66]. For instance, a rural population with only a primary education level had a consistently higher prevalence of hypertension than an urban population with higher educational levels [66]. In the analysis of data from global PURE that were related to socioeconomic status and risk for CVD in 20 low-, middle-, and high-income countries, education, rather than wealth, was the socioeconomic indicator most consistently associated with outcomes, and major CVD events and all-cause mortality were more common among people with low levels of education in all types of country studied. However, variances in outcomes among educational levels were not explained by variances in risk factors, which decreased with increasing educational level in high-income countries, but increased with increasing educational level in low-income countries [67]. Furthermore, results from the MONICA (Monitoring trends and determinants in cardiovascular disease) study suggested that a lower education level among the rural population could enhance CVD risk, but causality is difficult to prove [68].

In the present study, a greater proportion of individuals living in urban areas reported having an unhealthy diet, sadness, and stress. A recent global systematic evaluation of dietary consumption patterns across 195 countries found that improvement in diet prevents one in every five deaths globally and that a suboptimal diet is responsible for more deaths than other risk factors, including smoking, highlighting the urgent need to improve diet [69]. Urbanization is also associated with factors that could potentially influence mental health and possibly the development of CVD, such as increased life stressors, overcrowding, a higher level of violence, and less social support [70]. However, in addition to stress caused by a transition from a rural to urban area, other cultural factors interplaying with urban dynamics might contribute to the development of psychological-related problems. Therefore, understanding how cultural dynamics interact with adaptation to urban life may help in guiding appropriate management of mental disorders of people living in cities [71]. Awareness of the negative impact of urbanization on mental health is needed across Saudi society.

### Government recognition of the importance of the primary prevention of CVD diseases

Healthcare is a main focus of the Saudi Vision 2030, through which the Saudi government has initiated radical changes in the structure and function of its healthcare system via its National Transformation Program to achieve quality care and effective service delivery. In addition, the government has already recognized the importance of the primary prevention of CVD diseases and announced four major projects aimed at improving lifestyles [72–77]. Furthermore, the World Heart Federation has undertaken an initiative to develop a series of “roadmaps” to reduce premature deaths from CVD by at least 25% by 2025. These roadmaps can be used as guidance for countries seeking to develop or update their national non-communicable diseases programs for the prevention and control of these conditions [78].

### Limitations of the study

Our study has some limitations. First, the sampling framework of the PURE-Saudi study was not nationally representative, necessitating caution in generalizing the findings to the whole Saudi population. Additionally, relatively healthier individuals may have enrolled in our study; however; the prevalence of CVD risk factors is similar to that of previous national surveys, strengthening our study sampling methodology and results. Second, follow-up rates of CVD events and mortality were low, likely because of the small sample size. Efforts are ongoing to expand PURE Saudi to a larger population across all areas in the country to allow for meaningful event rates in the follow-up. Lastly, we cannot exclude the role of genetic predisposition in such a high prevalence of CVD risk factors, which could be related to high consanguinity in the Saudi population. We have recently reported a high prevalence of familial hypercholesterolemia in Saudi Arabia and the other Arabian Gulf countries [79].

## Conclusion

The PURE-Saudi study demonstrated that the continued high prevalence of unhealthy lifestyle and CVD risk factors in the adult Saudi population reflects a continued trend from a decade of several population surveys. Some of these factors were more prevalent in the rural than in the urban population. National awareness programs and multi-faceted healthcare policy changes are urgently needed to reduce the future burden of CVD risk and mortality.

## Supplementary information

**Additional file 1: Additional Table 1.** Risks of hyperlipidemia and hyperglycemia among men and women.

**Additional file 2: Additional Table 2.** Risks of hyperlipidemia and hyperglycemia according to age.

**Additional file 3: Additional Table 3.** Risks of hyperlipidemia and hyperglycemia in urban and rural populations.

**Additional file 4: Additional Table 4.** Characteristics of the PURE-Saudi study vs past studies that have assessed the prevalence of cardiovascular disease risk factors in Saudi Arabia.

## Data Availability

The data are not publicly available because the participants or the ethics committees have not given permission for sharing the data publicly and the study is ongoing.

## Abbreviations

CVD: Cardiovascular disease
PURE: Prospective Urban Rural Epidemiology
MET: Metabolic equivalent of task
PHCC: Primary healthcare center
SD: Standard deviation
IQR: Interquartile range
BMI: Body mass index
OHA: Oral hypoglycemic agent
SBP: Systolic blood pressure
DBP: Diastolic blood pressure
OPT: Occupied Palestinian Territory
UAE: United Arab Emirates
MONICA: Monitoring trends and determinants in cardiovascular disease
