# Supplementary material for: Demographic, behavioral, and cardiovascular disease risk factors in the Saudi population: results from the Prospective Urban Rural Epidemiology study (PURE-Saudi)
Source: BMC Public Health. 2020 Aug 8;20:1213. doi: 10.1186/s12889-020-09298-w (PMC7414714; doi:10.1186/s12889-020-09298-w)
Supplement: Supplementary file 1 — Additional file 1: Additional Table 1. Risks of hyperlipidemia and hyperglycemia among men and women. [file 12889_2020_9298_MOESM1_ESM.docx]

**Additional Table 1. Risks of hyperlipidemia and hyperglycemia according to age.**

HDL, high-density lipoprotein cholesterol; IQR, interquartile range; LDL, low-density lipoprotein cholesterol; SD, standard deviation

|  | **Total**  **N = 1775** | **35–49**  **n = 1171 (66)** | **50–59**  **n = 414 (23.3%)** | **60–70**  **n = 190 (10.7%)** | ***P*** |
| --- | --- | --- | --- | --- | --- |
| Total cholesterol, mean ± SD | 5 ± 1.2 | 5 ± 1 | 4.9 ± 1.2 | 4.9 ± 1.2 | 0.106 |
| Total cholesterol, median (IQR) | 4.9 (1.3) | 5 (1.3) | 4.9 (1.4) | 4.9 (1.6) | 0.135 |
| Fasting glucose, mean ± SD | 6.2 ± 2.9 | 5.7 ± 2.4 | 7.2 ± 3.5 | 7.6 ± 3.6 | <0.001 |
| Fasting glucose, median (IQR) | 5.2 (1.5) | 5 (1) | 5.9 (3) | 6.2 (3.2) | <0.001 |
| HDL cholesterol, mean ± SD | 1.1 ± 0.3 | 1.1 ± 0.3 | 1.2 ± 0.3 | 1.1 ± 0.3 | 0.049 |
| HDL cholesterol, median (IQR) | 1 (0.4) | 1.1 (0.4) | 1 (0.3) | 1 (0.4) | 0.068 |
| LDL cholesterol, mean ± SD | 3.2 ± 0.9 | 3.2 ± 0.9 | 3.1 ± 0.9 | 3.2 ± 1 | 0.080 |
| Triglycerides, median (IQR) | 1.3 (0.9) | 1.3 (0.9) | 1.3 (0.9) | 1.4 (0.8) | 0.001 |
| Fasting glucose between 6-7 mmol/l in nondiabetic patients, n (%) | 128 (9.5) | 73 (7.1) | 41 (17.6) | 14 (16.1) | <0.001 |
| Total cholesterol >5.2 mmol/l and LDL >3.5 mmol/l, n (%) | 569 (32.1) | 380 (32.4) | 124 (29.9) | 65 (34.2) | 0.514 |
