# Supplementary material for: Demographic, behavioral, and cardiovascular disease risk factors in the Saudi population: results from the Prospective Urban Rural Epidemiology study (PURE-Saudi)
Source: BMC Public Health. 2020 Aug 8;20:1213. doi: 10.1186/s12889-020-09298-w (PMC7414714; doi:10.1186/s12889-020-09298-w)
Supplement: Supplementary file 2 — Additional file 2: Additional Table 2. Risks of hyperlipidemia and hyperglycemia according to age. [file 12889_2020_9298_MOESM2_ESM.docx]

**Additional Table 2. Risks of hyperlipidemia and hyperglycemia among men and women.**

|  | **Women**  **n = 750 (42.2%)** | **Men**  **n = 1025 (57.7%)** | ***P*** |
| --- | --- | --- | --- |
| Total cholesterol, mean ± SD | 5 ± 1 | 4.9 ± 1 | 0.013 |
| Total cholesterol, median (IQR) | 5 (1.4) | 4.9 (1.3) | 0.022 |
| Fasting glucose, mean ± SD | 5.9 ± 2.6 | 6.5 ± 3.1 | <0.001 |
| Fasting glucose, median (IQR) | 5 (1.4) | 5.3 (1.7) | <0.001 |
| HDL cholesterol, mean ± SD | 1.2 ± 0.3 | 1 ± 0.3 | <0.001 |
| HDL cholesterol, median (IQR) | 1.2 (0.4) | 1 (0.3) | <0.001 |
| LDL cholesterol, mean ± SD | 3.2 ± 0.9 | 3.2 ± 0.9 | 0.698 |
| Triglycerides, median (IQR) | 1.2 (0.7) | 1.5 (1) | <0.001 |
| Fasting glucose between 6-7 mmol/l in nondiabetic patients, n (%) | 47 (7.9) | 81 (10.8) | 0.064 |
| Total cholesterol >5.2 mmol/l and LDL >3.5 mmol/l, n (%) | 234 (31.2) | 335 (32.7) | 0.508 |

HDL, high-density lipoprotein cholesterol; IQR, interquartile range; LDL, low-density lipoprotein cholesterol; SD, standard deviation
