# Supplementary material for: Demographic, behavioral, and cardiovascular disease risk factors in the Saudi population: results from the Prospective Urban Rural Epidemiology study (PURE-Saudi)
Source: BMC Public Health. 2020 Aug 8;20:1213. doi: 10.1186/s12889-020-09298-w (PMC7414714; doi:10.1186/s12889-020-09298-w)
Supplement: Supplementary file 3 — Additional file 3: Additional Table 3. Risks of hyperlipidemia and hyperglycemia in urban and rural populations. [file 12889_2020_9298_MOESM3_ESM.docx]

**Additional Table 3. Risks of hyperlipidemia and hyperglycemia in urban and rural population.**

|  | **Urban**  **n = 1394 (78.5%)** | **Rural**  **n = 381 (21.5%)** | ***P*** |
| --- | --- | --- | --- |
| Total cholesterol, mean ± SD | 5 ± 1.1 | 5 ± 1 | 0.900 |
| Total cholesterol, median (IQR) | 4.9 (1.3) | 5 (1.4) | 0.548 |
| Fasting glucose, mean ± SD | 6.2 ± 2.8 | 6.4 ± 3.3 | 0.307 |
| Fasting glucose, median (IQR) | 5.3 (1.4) | 5 (2.1) | 0.010 |
| HDL cholesterol, mean ± SD | 1.1 ± 0.3 | 1.1 ± 0.3 | 0.486 |
| HDL cholesterol, median (IQR) | 1 (0.4) | 1 (0.3) | 0.922 |
| LDL cholesterol, mean ± SD | 3.2 ± 0.9 | 3.2 ± 0.9 | 0.434 |
| Triglycerides, median (IQR) | 1.3 (0.9) | 1.3 (0.9) | 0.257 |
| Fasting glucose between 6-7 mmol/l in nondiabetic patients, n (%) | 113 (10.5) | 15 (5.6) | 0.014 |
| Total cholesterol >5.2 mmol/l and LDL >3.5 mmol/l, n (%) | 439 (31.5) | 130 (34.1) | 0.330 |

HDL, high-density lipoprotein cholesterol; IQR, interquartile range; LDL, low-density lipoprotein cholesterol; SD, standard deviation
