# Supplementary material for: Demographic, behavioral, and cardiovascular disease risk factors in the Saudi population: results from the Prospective Urban Rural Epidemiology study (PURE-Saudi)
Source: BMC Public Health. 2020 Aug 8;20:1213. doi: 10.1186/s12889-020-09298-w (PMC7414714; doi:10.1186/s12889-020-09298-w)
Supplement: Supplementary file 4 — Additional file 4: Additional Table 4. Characteristics of the PURE-Saudi study vs past studies that have assessed the prevalence of cardiovascular disease risk factors in Saudi Arabia. [file 12889_2020_9298_MOESM4_ESM.docx]

**Additional Table 4. Characteristics of the PURE-Saudi study vs past studies that have assessed the prevalence of cardiovascular disease risk factors in Saudi Arabia.**

| **Authors,**  **year** | **Age** | **Total sample**  **and**  **sex** | **Sampling method** | **Study design** | **Diagnostic criteria** | **Main findings (prevalence),**  **CVD risk** |
| --- | --- | --- | --- | --- | --- | --- |
| Al-Nuaim et al.  1997 | ≥20 y | 10,651  M: 50.8%  F: 49.2% | Multistage stratified cluster sampling | CSS | Overweight and obesity defined according to the WHO | Overweight  Overall: 31.2%  M 33.1%, F 29.4%  Obesity  Overall: 22.1%  M 17.8%, F 26.6% |
| Al-Nuaim et al.  1997 | 30–64 y | 2049  M: 1033  F: 1016 | Multistage stratified cluster sampling | CSS | DM: random serum glucose according to the WHO criteria or self-reported  HC: mild (5.2–6.2 mmol/l),  severe (>6.2 mmol/l) HDL: <0.9 mmol/l BMI: WHO criteria | Overweight: M 38%, F 34%  Obesity: M 23%, F 34%  DM: M 16.4%, F 20%  Smoking: M 21%, F 1%  Moderate HC: M and F 21.5%  Severe HC: M and F 9%  LDL: M 6.6%, F 10.3%  HDL: M 55%, F 47% |
| Abalkhail et al.  2000 | <35 y  35–39 y  >40 y | 1649  M: 71.3%  F: 28.7% | Random stratified sampling | CSS | Dyslipidemia:  TC: ≥240 mg/dl  HTN: SBP ≥140 or DBP ≥95 mmHg or on medication  Overweight: BMI ≥27.2 (men) and ≥26.9 kg/m^2^ (women) | Overweight: 49.8%  HTN: 19.9%  Current smoking: 18.8%  HC: 10.1% |
| Hashim et al.  2000 | 18–26 y | 647  M: 383  F: 264 | Random sampling | CSS | Current smokers:  Currently smoking at least 1 cigarette per day | Current smoking  Overall: 29%  M 20%, F 9% |
| Kalantan et al.  2001 | 35–85 y | 1114  M: 442  F: 672 | Cluster sampling | CSS | HTN: SBP ≥140 mmHg  and DBP ≥90 mmHg  and/or self-reported  with medication | HTN  Overall: 30%  M 33%, F 29% |
| Al-Refaee et al.  2001 | ≥19 y | 1333  M: 100% | Random sampling | CSS | Regular activity:  physically active for ≥30  minutes, ≥2 days a week | Physically inactive: 53%,  Irregularly active: 27.5%,  Physically active on a regular basis: 19% |
| Al-Haddad et al.  2003 | Mean 35.5 y | 1752 | Random sampling | CSS | Current smokers:  those who regularly or occasionally smoke on a daily, weekly, or monthly basis  Nonsmokers:  those who never smoked | Current  Smokers: 52.3% |
| Al-Nozha et al.  2004, 2005, 2007, 2008, 2009 | 30–70 y | 16,819–17,395 | Two-stage stratified cluster sampling | CSS | Diabetes, overweight, and obesity were defined according to the WHO  HTN: SBP ≥140  mmHg or DBP ≥90  mmHg  Physically active: ≥30  minutes of at least moderate intensity activity for ≥3 times per week.  Physical inactivity: participants who did not meet the physically active criteria  HC: TC ≥5.2 mmol/l TG: ≥1.69 mmol/l  History of smoking such as current, passive, or ex-smoking was obtained, as well as the type of tobacco smoked, quantity, and the duration of smoking | DM, Overall: 23.7%  M 26.2%, F 21.5%  IFG, Overall: 14.1%  M 14.4%, F 13.9%  Overweight, Overall: 36.9%,  M 42.4%, F 31.8%  Obesity, Overall: 35.6%,  M 26.4%, F 44%  HTN, Overall: 26.1%  M 28.6%, F23.9%  Physical inactivity, Overall: 96.1%  M 93.9%, F 98.1%  HC, Overall: 54%  M 54.9%, F 53.2%,  HG, Overall: 40.3%  M 47.6%, F 33.7%  Smoking, Overall: 12.8%  M 18.3%, F 7.3%  Smokers are more likely to develop CAD compared to non-smokers (*P* < 0.0001) |
| Al-Hamdan et al.  2005 | 15–64 y | 4758  M: 49.2%  F: 50.8% | Multistage stratified cluster random sampling/  Proposed methodology of STEPwise approach | CSS | STEPS Instrument for NCD risk factors as received from WHO, was used as basis for the study questionnaire | Physical inactivity: 67.6%  Obesity: 36.2%  Hypertension: 11.6%  Diabetes: 15.3%  HC: 19.1%  Current daily smoking: 10.9% |
| Al-Baghli et al.  2008 | ≥30 y | 195,874  M: 99,946  F: 95,905 | Non-random sampling | CSS | Overweight and  obesity defined  according to the WHO | Overweight, Overall: 35.1%  M 40.3%, F 29.7%  Obesity, Overall: 43.8%  M 36.1%, F 51.8% |
| Midhet et al.  2010 | 30–70 y | 2789  M: 1806  F: 981 | Random sampling | CSS | Not reported/questionnaire was used | The most popular food was  Kabsa: M 80%, F 65%  Fresh fruits: M 63%, F 45%  Vegetables: M 62%, F 47%  Dates: 45% both sexes  Soft drinks: M 21%, F 25% |
| El Bcheraoui et al  2014, 2105  Basulaiman et al.  2014  Memish et al.  2014  Moradi et al.  2015 | ≥15 y | 10,735 | Multistage Random sampling / The Saudi Health Information Survey (SHIS) | CSS | HTN: BP ≥140/90 mmHg  or taking medications for hypertension  Respondents were  classified as current, past, and never smoker based on self-reported data  Physical activity: International Physical Activity questionnaire  DM: HbA1c ≥6.5% or taking medication for diabetes  Obesity: BMI ≥30  Consumption of fruits and vegetables was measured using a frequency questionnaire according to CDC guidelines  Hypercholesterolemia:  LDL-c: ≥4.13 mmol/l  HDL-c: <1.0 mmol/l in M; <1.3 mol/l in F  TG: ≥2.3 mmol/l  Smoking status was assessed using 15 questions | HTN: 15.2%  Borderline hypertension: 40.6%  Undiagnosed hypertension: 57.8%  DM: 13.4%  Undiagnosed 57.8%,  Treated uncontrolled 20.2%,  Treated controlled 16.6%,  Untreated 5.4%  Met both the Saudi and the CDC guidelines for daily consumption of fruits and vegetables, Overall: 2.6%  Hypercholesterolemia,  Overall: 8.5%  Undiagnosed 65.1%, treated uncontrolled 2.3%, treated  controlled 28.3%,  untreated 4.3%  Low HDL: 48.7%  High LDL: 7.4%  High TG: 8.5%  Obese, Overall: 28.7%  M 24.1%, F 33.5%  Smoking, Overall: 12.2%  M 21.5%, F 1.1%  Daily shisha smoking, Overall: 4.3%  M 7.3%, F 1.3%  Secondhand smoking, Overall: 23.3%,  M 32.3%, F 13.5% |
| Al-Rubeaan et al.  2015 | ≥30 y | 18,034 | Random sampling | CSS | FPG: ≥126 mg/dl according to ADA criteria | DM, Overall: 25.4%  M 29.1%, F 21.9%  Unaware of their disease: 40.3%  IFG, Overall: 25.5%  M 26.4%, F 24.7% |
| Alzeidan et al.  2016 | Mean 39.3 y | 4500  M: 44.9%  F: 55.1% | A convenience sampling | CSS | DM was defined as per WHO and ADA criteria, or self-reporting  HTN was defined according to JNC7  Dyslipidemia according to the criteria of WHO and the National Cholesterol Education Program NCEP  Physically inactivity according to WHO criteria  Smoking: Current smoker, one cigarette daily for last 6 months | Obesity: 36%  Dyslipidemia: 22–37%  HTN: 22%  DM: 18%  Tobacco use: 12%  Physical inactivity: 77% |
| Ahmed et al.  2017 | Mean 43 y | 550  M: 71%  F: 29% | Random sampling | CSS | Dyslipidemia:  TC: ≥240 mg/dl  LDL-c: ≥100 mg/dl  HDL-c: <40 mg/dl in M; <50 mg/dL in F  TG: ≥200 mg/dl  HTN: BP ≥140/90 mmHg  DM: FBS ≥126 mg/dl  Obesity: BMI ≥30 kg/m^2^  Smoking, defined as current or past consumption of cigarettes, pipe, or water pipe (shisha) | Dyslipidemia: 68.6%  HTN: 41.8%  Obesity: 45.1%  DM: 25.9%  Smoking: 20% |
| Alharthi et al.  2017 | 20–40 y | 507  M: 76.3%  F: 23.7% | Non-probability convenience sampling | CSS | HTN: BP ≥140/90 mmHg  DM: RBS ≥200 mg/dl or FBS ≥126 mg/dl  Obesity: BMI ≥30 kg/m^2^ | HTN: 8.3%  DM: 0.6%  Overweight: 36.7%  Obesity: 29.6%  Smoking: 37.9% |
| Alhabib et al.  (PURE-Saudi) | 35–70 y | 2047  M: 56.9%  F: 43.1% | Random sampling of Riyadh and Al-kharj communities | Cohort | CVD risk factors as described in the INTERHEART study  Dyslipidemia:  TC: ≥5.2 mmol/l  HTN: BP ≥140/90 mmHg  or taking medications for hypertension  DM: FBS ≥126 mg/dl or taking medication for diabetes  Obesity: BMI ≥30 kg/m^2^  Physical activity was measured using the International Physical Activity Questionnaire | Physical inactivity: 69.4%  Obesity: 49.6%  Central obesity: 49.1%  Eating unhealthy diet: 34.4%  Dyslipidemia: 32.1%  HTN: 30.3%  DM: 25.1%  Current smoking: 12.2% |

M: male; F: female; y: years; CSS: cross-sectional study; DM: diabetes mellitus; HTN: hypertension; HC: hypercholesterolemia; TG: triglyceride; TC: total cholesterol; HDL: high-density lipoprotein; LDL: low-density lipoprotein; HTN: hypertension; SBP: systolic blood pressure; DBP: diastolic blood pressure; BMI: body mass index; WHO: World Health Organization; JNC7: Seventh Report of the Joint National Committee on Prevention, Detection, Evaluation, and Treatment of High Blood Pressure; ADA: American Diabetes Association; FPG: fasting plasma glucose; IFG: impaired fasting glucose; RBS: random blood sugar; NCEP: National Cholesterol Education Program; CDC: Centers for Disease Control and Prevention; NCD: non-communicable disease.
